# Ofloxacin Removal from Aqueous Media by Means of Magnetoactive Electrospun Fibrous Adsorbents

**DOI:** 10.3390/nano12203648

**Published:** 2022-10-18

**Authors:** Petri Ch. Papaphilippou, Oana Maria Marinica, Eugenia Tanasă, Fotios Mpekris, Triantafyllos Stylianopoulos, Vlad Socoliuc, Theodora Krasia-Christoforou

**Affiliations:** 1 Department of Mechanical and Manufacturing Engineering, School of Engineering, University of Cyprus, Nicosia 1678, Cyprus; 2 Department of Life Sciences, School of Science, European University Cyprus, Nicosia 2404, Cyprus; 3 Research Center for Engineering of Systems with Complex Fluids, Politehnica University Timisoara, 300222 Timisoara, Romania; 4 Faculty of Applied Chemistry and Materials Science, Politehnica University of Bucharest, 060042 Bucharest, Romania; 5 Laboratory of Magnetic Fluids, Center for Fundamental and Advanced Technical Research, Romania Academy—Timisoara Branch, 300223 Timisoara, Romania

**Keywords:** electrospinning, water remediation, ofloxacin, superparamagnetic iron oxide nanoparticles, magnetic fibrous adsorbents, nanocomposite fibers

## Abstract

Functionalized electrospun polymer microfibrous membranes were fabricated by electrospinning and further surface-functionalized with magnetic iron oxide (Fe_x_O_y_) nanoparticles to yield magnetoactive nanocomposite fibrous adsorbents. The latter were characterized in respect to their morphology, mechanical properties and magnetic properties while they were further evaluated as substrates for removing Ofloxacin (OFL) from synthetic aqueous media and secondary urban wastewater (UWW) under varying physicochemical parameters, including the concentration of the pharmaceutical pollutant, the solution pH and the membranes’ magnetic content. The magnetic-functionalized fibrous adsorbents demonstrated significantly enhanced adsorption efficacy in comparison to their non-functionalized fibrous analogues while their magnetic properties enabled their magnetic recovery and regeneration.

## 1. Introduction

During recent years, there has been a tremendous increase in the accumulation of pharmaceuticals in aquatic systems, owing to the exponentially increased amounts of drugs consumed worldwide [1,2]. Antibiotics such as Tetracycline, Ciprofloxacin, Levofloxacin, Amoxicillin, Norfloxacin, Ofloxacin, etc., predominate as organic pharmaceutical water contaminants [3]. Unfortunately, their unsuitable management and inconsiderable disposal into the ecosystem, including surface-, ground- and seawater, has led to severe environmental and health concerns, since such non-degradable compounds retain their stability and consequently can escape conventional activated sludge wastewater treatments [4,5]. Moreover, conventional technologies that are currently employed in sewage treatment plants, for example, exhibit low efficacy in the removal of such organic pollutants found at trace levels. As a result, the latter accumulate into aquatic systems and distribute into the environment [6]. Consequently, researchers worldwide have been focusing on the development of new and effective methods that could be employed in the removal of pharmaceutical and other contaminants from wastewater [7,8]. Among others, these are based on various processes, including coagulation, filtration, adsorption, sedimentation and photodegradation [9,10,11]. Of all the above, adsorption is considered to be one of the most efficient, simple, inexpensive and eco-friendly processes [12].

During the last two decades, electrospun polymer-based fibrous adsorbents characterized by high surface-to-volume ratios, tunable porosity, high permeability, good mechanical performance and tailor-made chemical composition and morphologies have attracted high attention in water-remediation processes for the removal of various contaminants from wastewater, including (radio)toxic metal ions [13,14,15], organic dyes [16,17], pharmaceutical compounds [18] and pathogenic microorganisms [19,20]. Electrospinning is a highly versatile nano- and microfiber production method that has already reached an industrial level [21,22] and can be employed in the fabrication of not only pure and blended polymer fibers but also ceramic and nanocomposite (nano)fibrous materials [23,24]. Consequently, the possibility provided of incorporating inorganic nanoparticulates that exhibit unique properties and functions within functional electrospun fibrous polymer matrices results in the generation of functionalized fibrous nanocomposites that could be further employed as highly effective adsorbents in water-remediation processes [25,26,27]. Among those, magnetic nanoparticles have been used as nanofillers in electrospun polymer-based fibrous membrane adsorbents, resulting in enhanced adsorption efficiency and providing the possibility for magnetic recovery by using an externally applied magnetic field. Examples include superparamagnetic fibrous adsorbents used in the removal of U(VI) [28], Eu(III) [29], Cr(VI) [30], arsenic ions [31], Congo red [26] and malachite green oxalate organic dyes [32], as well as antibiotics such as tetracycline [30,33], from synthetic aqueous media and urban wastewater (UWW). It was expected that the development of functionalized magnetic nanomaterials exhibiting high surface areas and high saturation magnetization such as magnetically functionalized graphene-based nanocomposites [34] would further promote the generation of highly effective magnetoactive adsorbents with potential to be used in water-remediation processes [35,36,37,38,39].

This study describes the development of blended electrospun polymer fibers consisting of an in-house synthesized random copolymer (poly(methyl methacrylate)-co-poly((2-(diethylaminoethyl) methacrylate), PMMA_x_-*co*-PDEAEMA_y_) prepared by free radical polymerization and a commercially available hydrophilic and crosslinkable polymer, namely poly(vinylpyrrolidone) (PVP). The resulting fibrous membranes were further surface-functionalized with magnetic iron oxide (Fe_x_O_y_) nanoparticles through a post-magnetization process involving the chemical co-precipitation of Fe(II) and Fe(III) salts under weak basic conditions in the presence of the fibrous membrane. The produced nanocomposite fibrous membranes were first characterized in respect to their morphology, mechanical properties and magnetic properties and further evaluated as adsorbents for the removal of ofloxacin (OFL) from synthetic aqueous media and secondary UWW. This antibiotic—belonging to the general class of fluoroquinolones—is frequently used in the treatment of urinary and respiratory infections. OFL adsorption efficiency was valuated as a function of various physicochemical parameters, including OFL initial solution concentration, pH and magnetic content. The magnetic-functionalized fibrous adsorbents demonstrated significantly improved adsorption efficacy for OFL compared to the pristine, magnetic-free fibrous analogues, as well as the possibility for magnetic recovery and regeneration.

## 2. Materials and Methods

### 2.1. Chemicals and Reagents

Methyl methacrylate (MMA, Fluka, AG, Buchs, Switzerland, ≥99%) and 2-(diethylaminoethyl) methacrylate (DEAEMA, Sigma–Aldrich, St. Louis, MI, USA, 99%) were passed through a basic alumina column (Activated, basic, Brockmann I, ~150 mesh, pore size 58 Å, Sigma–Aldrich, St. Louis, MI, USA) prior to the polymerization reactions. The radical initiator 2,2′-azobis(2-methylpropionitrile) (AIBN, Sigma–Aldrich, St. Louis, MI, USA, 95%) was recrystallized twice from ethanol. Polyvinylpyrrolidone (PVP) (Mn~1,300,000, Sigma–Aldrich, St. Louis, MI, USA) was used as received by the manufacturer. N-hexane (Sharlau, Barcelona, Spain 96%), tetrahydrofuran (THF, Sharlau, Barcelona, Spain extra pure), ethyl acetate (EA, GC grade, Sigma–Aldrich, St. Louis, MI, USA, 99.8%) and ammonium hydroxide (Sigma–Aldrich, St. Louis, MI, USA, 25% (*v*/*v*) in H_2_O) were used without further purification. Deuterated chloroform (Merck), methanol (Sharlau, Barcelona, Spain, Analytical grade, ACS reagent), hydrochloric acid (HCl, Merck, Kenilworth, New Jersey, USA, 37%), sodium hydroxide pellets (NaOH, Scharlau, Barcelona, Spain), iron chloride(III) hexahydrate (FeCl_3_.6H_2_O, Sigma–Aldrich, St. Louis, MI, USA, 97%) and iron chloride(II) tetrahydrate (FeCl_2_.4H_2_O, Sigma–Aldrich, St. Louis, MI, USA, 99%) were used as provided by the manufacturer. Ofloxacin (OFL, Sigma–Aldrich, St. Louis, MI, USA) was used without further treatment. OFL aqueous solutions were prepared in deionized water (pH = 6.0−6.5).

### 2.2. Polymer Synthesis

A random copolymer consisting of MMA and DEAEMA units, denoted as PMMA_x_-*co*-PDEAEMA_y_, was synthesized by conventional free radical polymerization as follows: Initially, MMA (9.43 g, 94.2 mmol) and DEAEMA (11.6 g, 62.8 mmol) were transferred in a round-bottom flask (100 mL) with the aid of a glass syringe. Subsequently, AIBN (0.26 g, 1.57 mmol) dissolved in EA (5 mL) as well as an additional amount of EA (20 mL) were also added into the flask. The reaction mixture was then placed in an oil bath and stirred at 65 °C for 24 h under a dry nitrogen atmosphere. Finally, the reaction mixture was left to cool down to room temperature and the produced copolymer was precipitated in n-hexane to yield PMMA_x_-*co*-PDEAEMA_y_ random copolymer (10.6 g, 50% polymerization yield), a white colored solid. Eventually, the produced copolymer was placed in a vacuum oven to dry (24 h, 25 °C).

^1^H-NMR (CDCl_3_, δ, ppm): 4.02 (2H, s, CH_2_), 3.59 (3H, s, CH_3_), 2.71 (2H, s, CH_2_), 2.56–2.62 (4H, br, CH_2_), 1.80–1.94 (2H, br, CH_2_), 1.72 (2H, br, CH_2_), 1.01–1.08 (3H, br, CH_3_) and 0.85 (3H, s, CH_3_).

### 2.3. Membrane Fabrication

**Figure 1:**
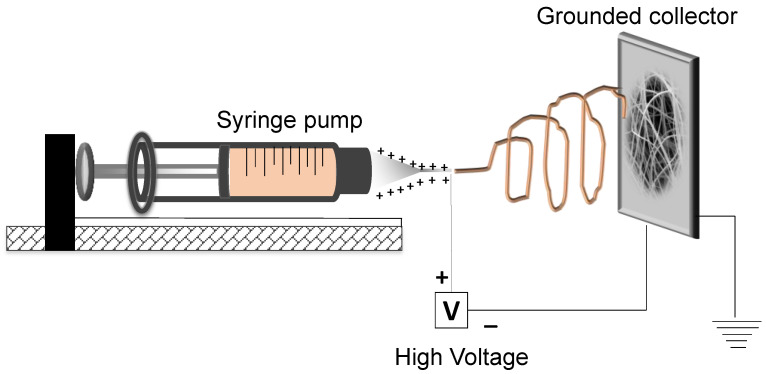
Schematic representation of the electrospinning setup.

A single-nozzle, custom-made electrospinning setup, which is schematically depicted in Figure 1, was used in the fabrication of fibrous membranes consisting of blended PMMA_x_-co-PDEAEMA_y_/PVP electrospun polymer fibers. The electrospinning experiments were carried out at room temperature (25 °C) and under ~35–40% humidity levels. For the production of the PMMA_x_-*co-*PDEAEMA_y_/PVP electrospun fibrous membranes, a homogeneous solution containing PMMA_x_-*co*-PDEAEMA_y_ (0.75 g) and PVP (0.75 g) (i.e., a 1:1 wt. polymer mixture) was initially prepared in CHCl_3_ (5 mL) at a 30% *w*/*v* polymer solution concentration upon stirring for 24 h at room temperature. Afterwards, the resulting homogeneous PMMA_x_-*co-*PDEAEMA_y_/PVP solution was transferred into a 10 mL glass syringe connected with a metallic needle (16 G) and the flow rate was set at 2.5 mL·h^−1^ using a flow controller (KDS 789252, KD Scientific Inc., Holliston, Massachusetts, USA. The applied voltage and distance between the tip of the needle and the stainless-steel grounded collector were set at 20 kV and 15 cm, respectively. The PMMA-co-PDEAEMA/PVP fibrous membrane was then thermally crosslinked upon heating at ca. 180 °C for 5 h, thus rendering it insoluble in aqueous media.

The magnetic functionalization of the crosslinked PMMA-co-PDEAEMA/PVP fibrous membrane was carried out by the chemical co-precipitation of Fe(III) and Fe(II) cations in a 2:1 molar ratio under weak basic conditions. Prior to the reaction, deionized water and ammonium hydroxide solution (Sigma–Aldrich, St. Louis, MI, USA, 25% (*v*/*v*) were purged with high-purity N_2_ for 30 min to remove oxygen. The reaction was performed under continuous nitrogen flow.

A typical experimental procedure is described as follows: Initially, (0.147 g, 0.54 mmol) FeCl_3_.6H_2_O was transferred into a glass vial (20 mL). Degassed, deionized water (3 mL) was subsequently added, followed by the addition of FeCl_2_.4H_2_O (0.0537 g, 0.27 mmol) dissolved in degassed, deionized water (3 mL). The resulting mixture was left to stir under an inert nitrogen atmosphere for 15 min, resulting in an orange-colored, transparent and homogeneous solution. In the meantime, the PMMA_x_-*co-*PDEAEMA_y_/PVP crosslinked fibrous membrane (32 mg) was fully immersed in a degassed aqueous solution (6 mL). Afterwards, the Fe(III)/Fe(II) aqueous solution was transferred with a syringe to the membrane-containing solution, followed by stirring for 15 min before the dropwise addition of the ammonium hydroxide solution (0.36 mL) while purging with extra-pure N_2_ for an additional 30 min. During this time, the solution immediately turned from homogeneous orange-colored to non-homogeneous dark-brown-colored, indicating the formation of iron oxide (Fe_x_O_y_) nanoparticles. The magnetically functionalized PMMA_x_-*co-*PDEAEMA_y_/PVP-Fe_x_O_y_ fibrous membrane was then collected by magnetic separation and washed several times with deionized water to remove the unbound Fe_x_O_y_ nanoparticles and unreacted products. The membrane was then dried at 40 °C under vacuum for 24 h.

### 2.4. Membrane Characterization

Molecular characterization of the PMMA_x_-*co*-PDEAEMA_y_ random copolymer was carried out by size exclusion chromatography (SEC) supplied by PSS Polymer Standards Service GmbH (Mainz, Germany) and nuclear magnetic resonance (^1^H NMR) spectroscopy (Avance Brucker 500 MHz spectrometer, Bruker, Billerica, MA, USA).

SEC was used to determine the average molar mass (MM) and molar mass distribution (MMD) of the PMMA_x_-*co*-PDEAEMA_y_ random copolymer, using equipment supplied by Polymer Standards Service (PSS). All measurements were carried out at room temperature using Styragel HR 3 and Styragel HR 4 columns. THF was used as a mobile phase (flow rate: 1 mL min^−1^). A Waters 515 isocratic pump was used for this purpose while the refractive index was measured with a Waters 2414 refractive index detector. Poly(methyl methacrylate) (PMMA) standards with a low polydispersity index (PDI; MWs of 739,000, 446,000, 270,000, 126,000, 65,000, 31,000, 14,400, 4200, 1580, 670, 450 and 102 (methyl isobutyrate) g mol^−1^), supplied by PSS, were used in system calibration.

The ^1^H NMR spectrum of the PMMA_x_-*co*-PDEAEMA_y_ random copolymer was recorded in CDCl_3_ with tetramethylsilane (TMS) used as an internal standard, using an Avance Bruker 500 MHz spectrometer (Bruker, Billerica, MA, USA) equipped with an Ultrashield magnet.

The morphology of the produced membranes was analyzed by scanning electron microscopy (SEM) (Vega TS5136LS-Tescan). All samples were gold-sputtered (~100 nm) (K575X Turbo Sputter Coater-Emitech, Quorum Technologies Ltd., West-Sussex, UK) prior to SEM analysis for reduction of the effect of surface charging. Transmission electron microscopy (TEM) was utilized to visualize the iron oxide (Fe_x_O_y_) nanoparticles that were anchored onto the fibers’ surfaces. For this purpose, a TECNAI F30 G2 S-TWIN microscope operating at 300 kV and equipped with an energy-dispersive X-ray spectrometer (EDX) (FEI Company, the Netherlands) was used while samples were placed into a double copper grid (oyster).

Tensile experiments were performed using a high-precision mechanical testing system (Instron 5944, Norwood, MA, USA). Orthogonal specimens were prepared with dimensions of 5.0 × 6.0 × 1.0 mm (length × width × thickness) and were held by two grips. Stress–strain experiments were performed to measure the elastic behavior of the material. The specimens were stretched to 25% strain with a strain rate of 0.5 mm/min. The stress was calculated as the force measured on the load cell divided by the initial area of the specimen (i.e., 1st Piola–Kirchhoff stress) and the strain was calculated as the displacement ∆l divided by the initial length of the specimen. The Young’s modulus was calculated from the slope of the linear part of the stress–strain curves for low strains (< 5%). Six specimens were tested (*n* = 6).

The X-ray diffraction pattern of the magnetically functionalized membrane was obtained using Rigaku (30 kV, 25 mA) with *λ* = 1.5405 Å (Cu). The magnetic properties of the produced magnetically functionalized fibrous adsorbent was measured by vibrating sample magnetometry (VSM) using an ADE Technologies VSM880 magnetometer (ADE Technologies inc., Lowell, MA, USA). The measurement was performed at room temperature, in the magnetic field intensity range of −1000 kA/m–1000 kA/m.

### 2.5. Adsorption Studies

#### 2.5.1. Adsorption Studies in Synthetic Aqueous Media

To evaluate the performance of the produced electrospun PMMA_x_-*co-*PDEAEMA_y_/PVP (pristine) and PMMA_x_-*co-*PDEAEMA_y_/PVP-Fe_x_O_y_ (magnetoactive) fibrous membranes in the adsorption of OFL from synthetic aqueous solutions, batch adsorption experiments were performed under ambient conditions. All experiments were performed in glass vials (20 mL) with a plastic snap-cap.

OFL adsorption kinetic studies were performed by UV–vis spectrophotometry (Jasco V-630, Jasco Corporation, Tokyo, Japan) at room temperature. OFL was dissolved in deionized water (solution concentration: 0.1 g·L^−1^), and the resulting solution was used as stock solution (solution pH = 6.8). OFL aqueous solutions of various concentrations (0.78–18.75 mg·L^−1^) were obtained upon diluting the stock solution with deionized water.

**Figure 2:**
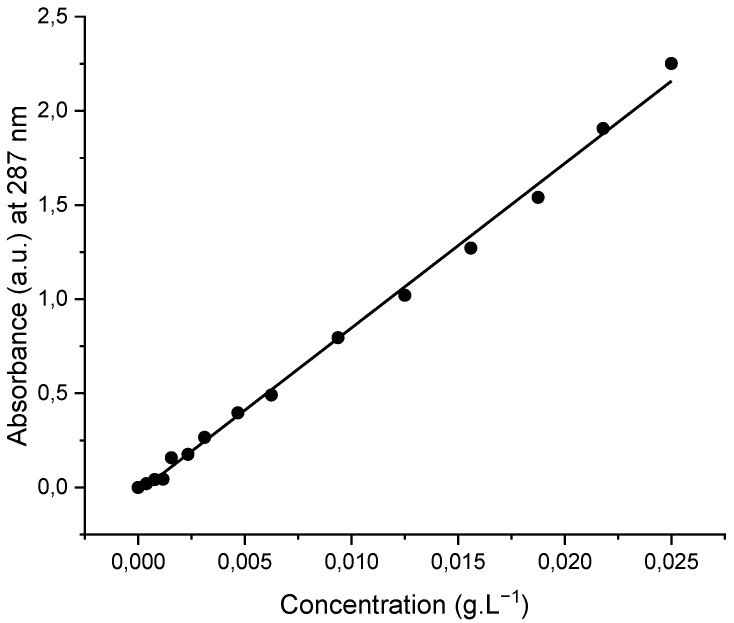
Calibration curve of OFL recorded in deionized water.

For investigating the effect of pH on the membranes’ adsorption performance, solution pH was adjusted by adding 0.01 M and/or 0.05 M aqueous HCl solution. Subsequently, a dried membrane sample (10 mg) was immersed in an OFL aqueous solution (5 mL, initial concentration of 0.01875 g·L^−1^) prepared at different pH values (pH = 4.0 and pH = 6.0). At specific time intervals, an aliquot was extracted from the solution and placed in the UV–vis spectrophotometer for recording the UV–vis spectrum of the supernatant solution containing OFL (adsorption wavelength: 287 nm). Upon the completion of each measurement, the aliquot was returned back into the vial. The OFL removal efficiency (%) was calculated using an absorbance (recorded at 287 nm) versus concentration (g·L^−1^) calibration curve (correlation coefficient, R^2^ = 0.996) (Figure 2). All the adsorption experiments were performed in triplicate for each system in order to verify the repeatability of the measurements.

For investigating the effect of the initial OFL concentration, a specific amount of the magnetoactive fibrous adsorbent (10 mg) was placed in aqueous solutions (5 mL) prepared in DI water containing various OFL concentrations (0.78, 2.34, 6.25 and 12.5 mg·L^−1^). In all cases, the pH value was adjusted to 4. The UV–vis spectrum of the supernatant solution was then recorded to evaluate the amount of the adsorbed OFL, denoted as adsorption capacity, *q_e_* (mg·g^−1^), by measuring the absorbance at 287 nm. The latter as well as the % removal efficiency (% *q_e_*) were calculated using the following equations: [40]

(1) $$q_{e}=\frac{C_{0}-C_{aq}}{W}\cdot V$$

(2) $$q_{e}\%=\frac{C_{0}-C_{aq}}{C_{0}}\cdot 100$$

where *q_e_* (mg·g^−1^) is the adsorbed amount of OFL, *C*_0_ (mg·L^−1^) and *C_aq_* (mg·L^−1^) are the initial and equilibrium concentrations of the drug in solution, *V* (L) is the volume of OFL solution and *W* (mg) is the weight of the dry fibrous adsorbent.

#### 2.5.2. Ofloxacin Removal from Urban Wastewater

A solution containing OFL (concentration: 2.34 mg·L^−1^) was prepared by spiking the appropriate mass of the compound into the secondary treated effluent sample collected from the Urban Wastewater (UWW) treatment plant located on the premises of the University of Cyprus. The incorporation of a higher quantity of the OFL antibiotic into the UWW sample than that typically found in real wastewater samples was preferred in order to enable the detection and measurement of residual OFL by employing typical analytical methods. Solution pH was adjusted at ~ 4 by using an HCl aqueous solution (0.05–0.01 M). A specific amount of the dried magnetoactive fibrous membrane (10 mg) was immersed into the solution and aliquots were withdrawn at various time intervals and further analyzed by UV–vis spectrophotometry to record the characteristic absorbance signal of OFL appearing at 287 nm.

### 2.6. Desorption Studies

Desorption studies were performed at various temperatures (25, 37 and 60 °C) by immersing the OFL-containing magnetoactive fibrous adsorbent (10 mg) in alkali solution (5 mL) (NaOH 0.005 M–0.01 M) at pH 8.5, followed by the removal of the supernatant solution at specific time intervals. UV–vis spectrophotometry was used to record the optical density of the collected solution at 287 n so as to determine the concentration of the desorbed OFL. The desorption (%) of OFL was determined based on Equation (3):

(3) $$Desorption\;\left(\%\right)=\frac{C_{OFL,\;desorbed}}{C_{OFL,\;total\;adsorbed}}*100$$

## 3. Results

### 3.1. Polymer Synthesis and Molecular Characterization

A PMMA_x_-*co*-PDEAEMA_y_ random copolymer comprising both hydrophilic/cationic (DEAEMA) and hydrophobic (MMA) units was successfully synthesized by conventional free radical polymerization.

**Figure 3:**
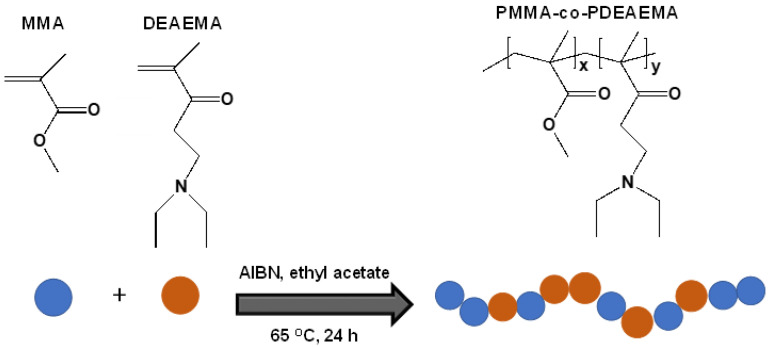
Synthetic procedure followed for the preparation of the PMMA_x_-*co*-PDEAEMA_y_ random copolymer by free radical polymerization and chemical structures of the MMA and DEAEMA monomeric units.

The polymerization methodology followed, as well as the chemical structures of the monomers (MMA, DEAEMA) and the initiator (AIBN), are provided in Figure 3. SEC was employed to determine the number average molar mass (M_n_ = 69,104 g·mol^−1^) and molar mass distribution (MMD = 2.60) of the PMMA_x_-*co*-PDEAEMA_y_ copolymer. As expected, the polymer possessed a relatively high PDI due to the non-controlled character of the free radical polymerization process [41]. Moreover, the copolymer chemical composition was evaluated using ^1^H NMR spectroscopy. More specifically, the molar ratio of the two repeating units (MMA, DEAEMA) incorporated within the copolymer chain was determined to be 1:0.7, respectively, by assigning the characteristic resonance peaks appearing in the ^1^H NMR spectrum, corresponding to each one of the two monomer units.

### 3.2. Membrane Fabrication and Characterization

#### 3.2.1. Membrane Fabrication and Morphological Characterization

**Figure 4:**
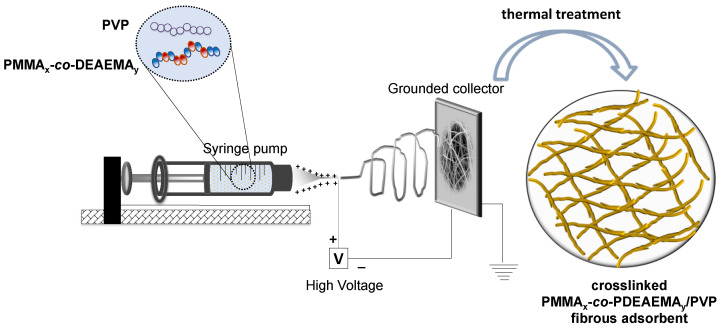
Schematic representation of the electrospinning process employed in the preparation of the PMMA_x_-*co*-PDEAEMA_y_/PVP blended fibrous membrane that was further crosslinked via thermal treatment.

Electrospinning was employed to fabricate blended fibrous membranes consisting of PMMA_x_-*co-*PDEAEMA_y_ and PVP, as schematically shown in Figure 4. Through employment of the optimum electrospinning conditions (polymer solution concentration: 30 *w*/*v*; applied voltage: 20 kV; needle gauge: 16 G; needle-to-collector distance: 15 cm; flow rate: 2.5 mL·h^−1^), the PMMA_x_-*co-*PDEAEMA_y_/PVP electrospun fibrous membrane was successfully produced. In order to render the as-prepared membrane insoluble in water, a thermal treatment process was employed. Based on a previous study by our group, [42] an FTIR analysis performed on thermally crosslinked PVP-containing electrospun fibrous membranes revealed the existence of small changes appearing around 1250 cm^−1^, which was assigned to C-N stretching and the bands at 880 cm^−1^, corresponding to the breathing vibration of the pyrrolidone ring and thus indicating the success of the PVP crosslinking process. The latter was further supported by the fact that the thermally crosslinked membranes were insoluble in aqueous media, in contrast to the non-crosslinked analogues.

**Figure 5:**
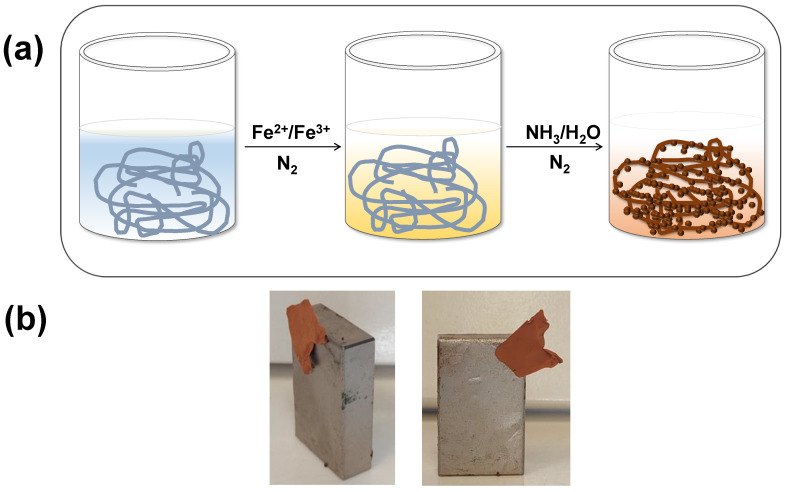
Schematic of the post-magnetization process followed for the preparation of the magnetically functionalized PMMA_x_-*co-*PDEAEMA_y_/PVP-Fe_x_O_y_ electrospun fibrous adsorbents (**a**); photograph demonstrating the attraction of the membrane by an external magnet (**b**).

The post-magnetization process employed in the preparation of the magnetoactive PMMA_x_-*co-*PDEAEMA_y_/PVP-Fe_x_O_y_ electrospun fibrous membrane involved the chemical co-precipitation of Fe(II) and Fe(III) under alkaline conditions [43]. By performing the above-mentioned chemical reaction in the presence of the PMMA_x_-*co-*PDEAEMA_y_/PVP fibrous membrane, the magnetic iron oxide nanoparticles produced were anchored onto the fibers’ surfaces, as schematically presented in Figure 5a. Moreover, as seen in Figure 5b, the resulting brown-colored magnetically functionalized membrane could be attracted by a permanent magnet, thus providing the possibility of its removal from aquatic systems by applying an external magnetic field. According to Huang and co-workers, tertiary amino-functionalities are capable of binding onto the surfaces of iron oxide NPs, which justifies the effective anchoring of the in situ synthesized Fe_x_O_y_ NPs onto the surfaces of the DEAEMA-containing fibers [44].

**Figure 6:**
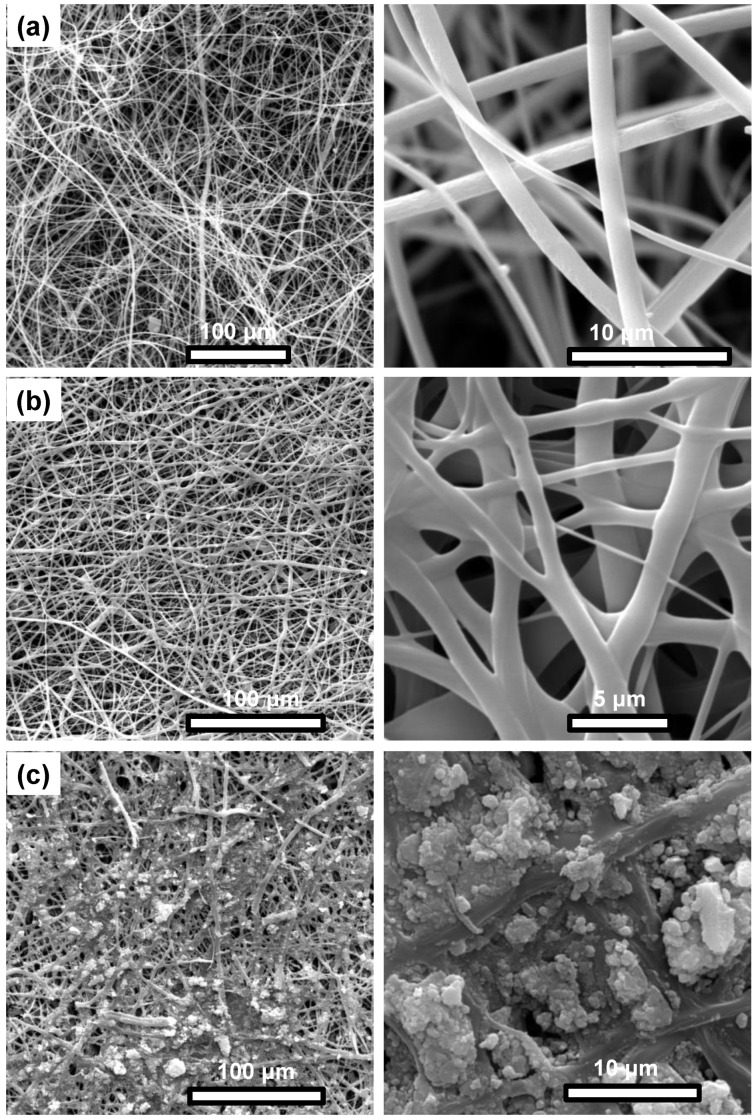
Representative SEM images of PMMA_x_-co-PDEAEMA_y_/PVP (as-prepared) (**a**), PMMA_x_-co-PDEAEMA_y_/PVP (crosslinked) (**b**) and PMMA_x_-*co*-PDEAEMA_y_/PVP/Fe_x_O_y_ (magnetically functionalized) electrospun membranes (**c**).

SEM was employed to obtain information on the morphology of the produced materials. Figure 6 provides characteristic SEM images of the as-prepared, non-crosslinked PMMA_x_-co-PDEAEMA_y_/PVP fibers (Figure 6a), the corresponding crosslinked fibers (Figure 6b) and the surface-functionalized PMMA_x_-*co*-PDEAEMA_y_/PVP/Fe_x_O_y_–crosslinked fibers (Figure 6c). As seen, the produced fibrous membranes obtained under the optimum electrospinning conditions consisted of continuous, bead-free and cylindrical fibers with smooth surfaces. Their average diameters were determined to be 1.055 ± 0.440 μm. However, upon thermal crosslinking, partial merging of the fibers was observed, accompanied by an increase in their average diameters and decrease in homogeneity (7.005 ± 5.975 μm). This phenomenon might be attributed to the one-step thermal treatment process employed at 180 °C. This effect can be diminished by following a milder thermal treatment protocol involving a three-step heating process [42].

In the case of the post-magnetized fibrous membrane, the presence of Fe_x_O_y_ NP aggregates on the membrane’s surface could be clearly observed. Furthermore, although the morphology and average diameters of the fibers remained relatively unaffected, upon fiber hydration during the chemical co-precipitation process, some morphological changes could be observed, i.e., partial fiber swelling, in line with previous studies [45,46].

TEM was also employed to study the morphology of the magnetically functionalized fibrous adsorbents.

**Figure 7:**
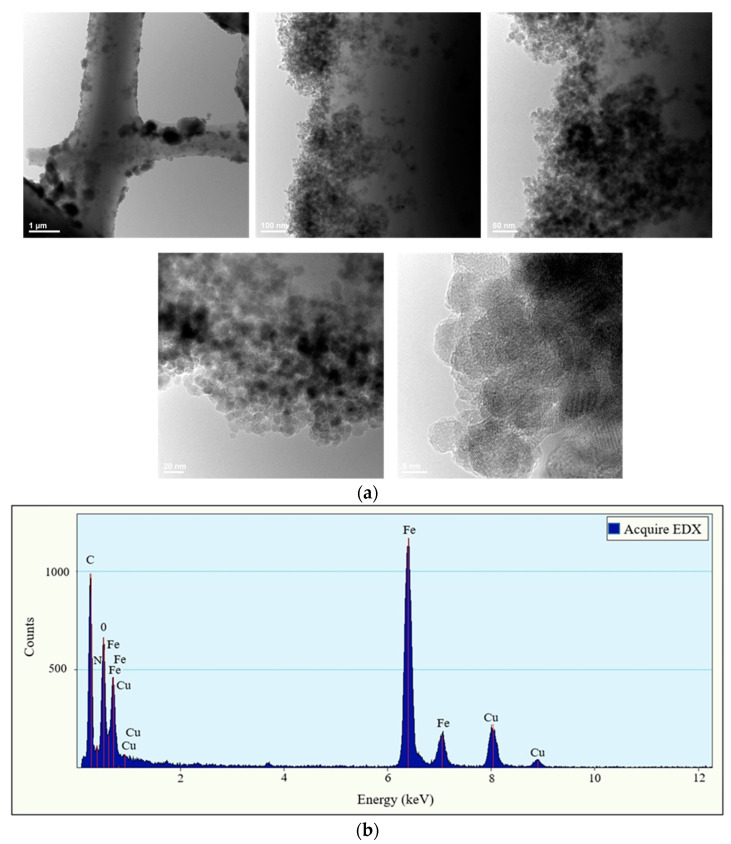
TEM bright-field images (**a**) and EDX spectrum of the magnetically functionalized electrospun membranes (**b**).

As seen in the TEM bright-field images provided in Figure 7a, Fe_x_O_y_ NP aggregates could be observed on the fibers’ surfaces, indicating their successful anchoring onto the fibers during the post-magnetization step, in agreement with our previous study involving the post-magnetization of chitosan-based electrospun nanofibers [43]. Furthermore, the EDX spectrum provided in Figure 7b shows the presence of Fe, O, N and C as the major elements in the sample (element Cu comes from the copper grid).

#### 3.2.2. Determination of the Fe_x_O_y_ Nanocrystalline Phase

**Figure 8:**
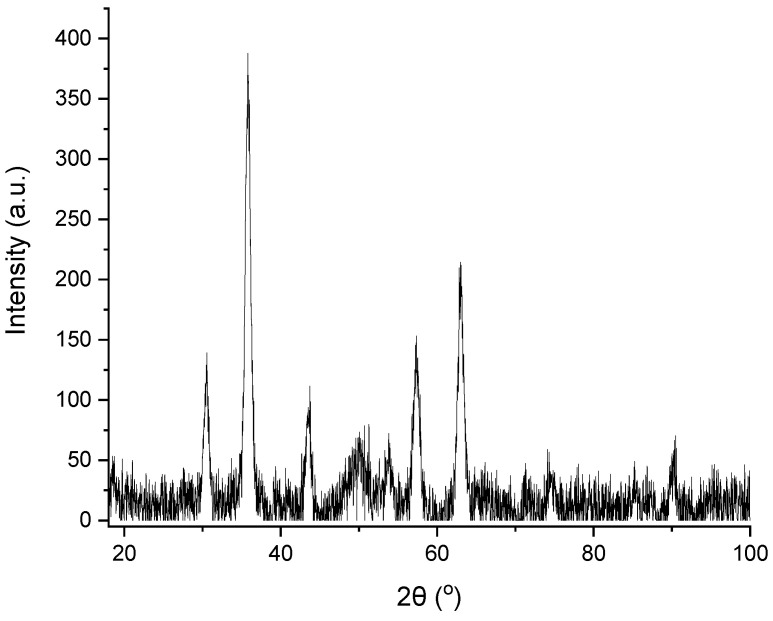
X-ray diffraction pattern of the magnetically functionalized electrospun fibrous adsorbent, denoting the presence of Fe_3_O_4_ NPs.

XRD was employed to determine the nanocrystalline phase adopted by the Fe_x_O_y_ NPs that were generated in situ and simultaneously deposited onto the surfaces of the fibrous adsorbent. Figure 8 provides the XRD diffraction pattern of the magnetically functionalized electrospun nanocomposite membrane. Six broad peaks appear at 2θ∼30°, 36°, 43°, 54°, 58° and 63°, indicating the presence of Fe_3_O_4_ NPs, in agreement with previously reported studies [47,48,49,50].

#### 3.2.3. Magnetic Properties

**Figure 9:**
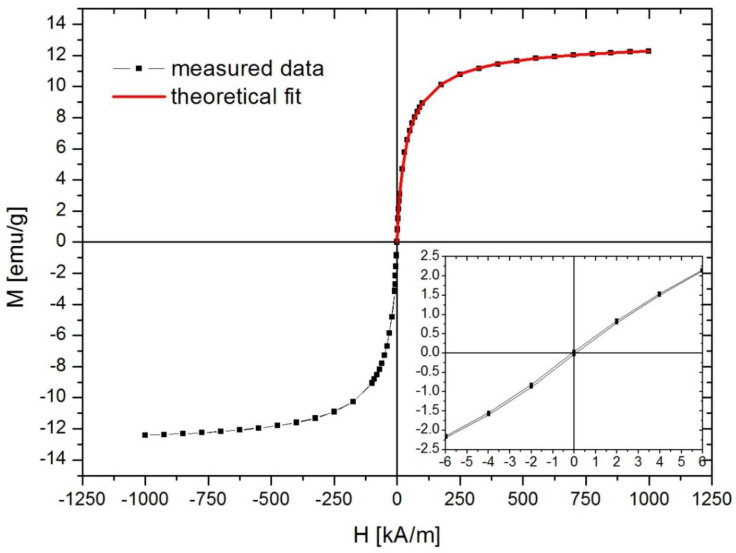
Magnetization (M) versus applied magnetic field strength (H) plot corresponding to the PMMA_x_-co-PDEAEMA_y_/PVP/Fe_x_O_y_ magnetoactive electrospun fibrous adsorbent.

Figure 9 illustrates the magnetic hysteresis (M vs. H) curve obtained at 300 K. The sample is superparamagnetic with negligible remanence and coercivity (M_r_ = 0.035 emu/g, H_c_ = 0.08 kA/m). The saturation magnetization of the sample, measured at 1000 kA/m, was 12.3 emu/g, which indicated a high degree of magnetic loading. Using the magnetite saturation magnetization 93 emu/g, the magnetic loading of the sample was calculated to be 13.2 wt.%. From the data fit (R^2^ = 0.99998) with a magnetization theoretical model, [51] the iron oxide nanoparticles’ magnetic diameter was found to be 5.4 ± 2.3 nm. The fit curve is presented in Figure 8. According to Tolmacheva et al., [52] hypercrosslinked polystyrene (HCPS) and Fe_3_O_4_ nanoparticles (HCPS–Fe_3_O_4_) adsorbents were tested for the removal of various tetracycline antibiotics separation from aqueous media. In those systems, Ms was found to range between 1 and 10 emu·g^−1^, which was found to be in good agreement with the value recorded in the present study.

#### 3.2.4. Mechanical Properties

**Figure 10:**
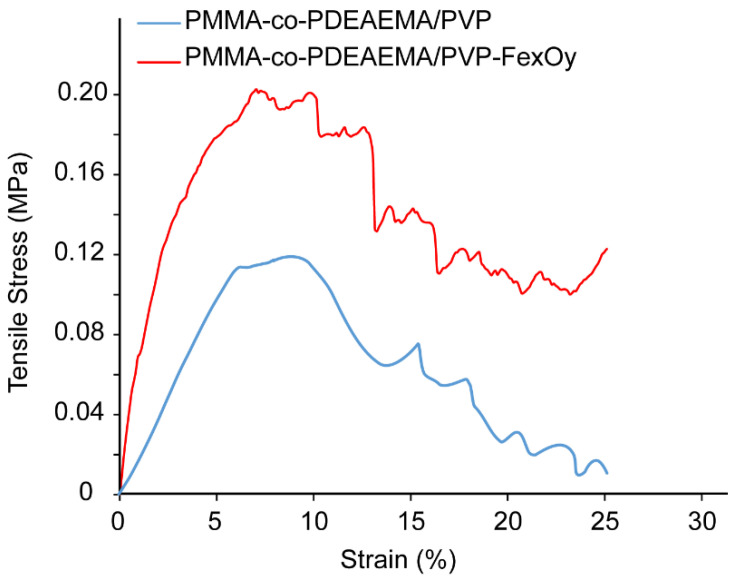
Stress–strain curves recorded under tensile conditions for the pristine membrane (blue) and the magnetically functionalized electrospun fibrous membrane (red).

The mechanical behavior of the pristine and magnetically functionalized crosslinked electrospun fibrous membranes was investigated under tensile loading conditions. Representative stress–strain curves for each case are provided in Figure 10. The Young’s modulus for each membrane was calculated from the slope of the linear part of the stress–strain curves for low strains (<5%). The corresponding average values were 2.09 ± 1.09 MPa for pristine and 4.56 ± 1.86 MPa for magnetoactive membranes; the difference is statistically significant. Furthermore, a yield point was observed at ~7% strain for both membrane types whereas the magnetically functionalized membranes exhibited a higher yield stress, i.e., 0.12 ± 0.03 MPa for pristine and 0.23 ± 0.05 MPa for magnetoactive membranes, with statistically significant difference. The mechanical enhancement observed in the case of the magnetically functionalized membrane might be attributed to the presence of the Fe_x_O_y_ NPs onto the fibers’ surfaces, which may have acted as crosslinking points among the PVP chains (through the development of coordination bonds with the C=O group) and the DEAEMA moieties (through the tertiary amino functionalities) existing on the fibers’ surfaces [53,54].

### 3.3. Adsorption Studies

#### 3.3.1. Ofloxacin Removal from Synthetic Aqueous Media

The investigation of OFL removal from synthetic aqueous media in the presence of either the pristine or the magnetically functionalized electrospun fibrous adsorbent was carried out by conducting batch-type experiments. UV–vis spectrophotometry was used to monitor the adsorption process.

**Figure 11:**
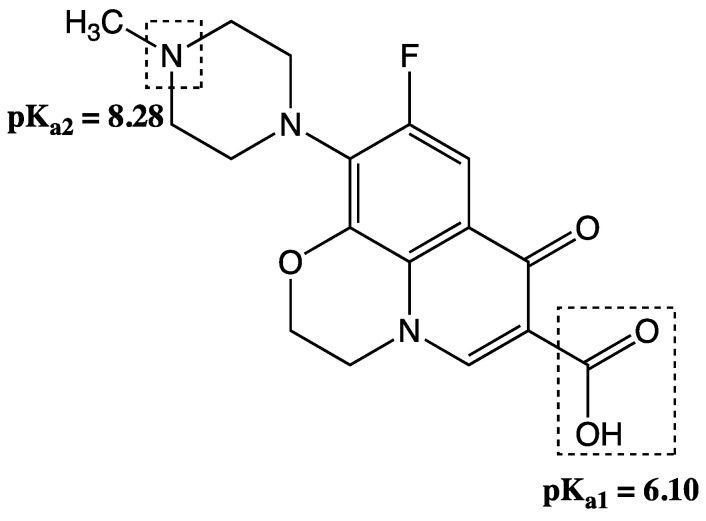
Chemical structure of OFL. The pH responsive moieties (3-carboxyl and piperazinyl groups) are denoted in dashed squares.

pH is one of the most important parameters that govern adsorption efficiency [55,56,57,58]. In the present study, solution pH may have strongly affected the membranes’ removal efficiency, since OFL possesses several functional groups that are influenced by pH. More precisely, OFL has two ionizable functional groups and thus exhibits two different pK_a_ values, as depicted in Figure 11. The 3-carboxyl group presented a pk_a_ equal to 6.10 and the nitrogen atom of piperazinyl group a pk_a_ equal to 8.28. Consequently, at pH < 6.1, the cationic form dominates whereas at pH > 8.28, the anionic form exists. Within the pH range of 6.1–8.28, OFL exists partially in both the zwitterionic (OFL^±^) and the neutral (OFL^0^) form [59].

**Figure 12:**
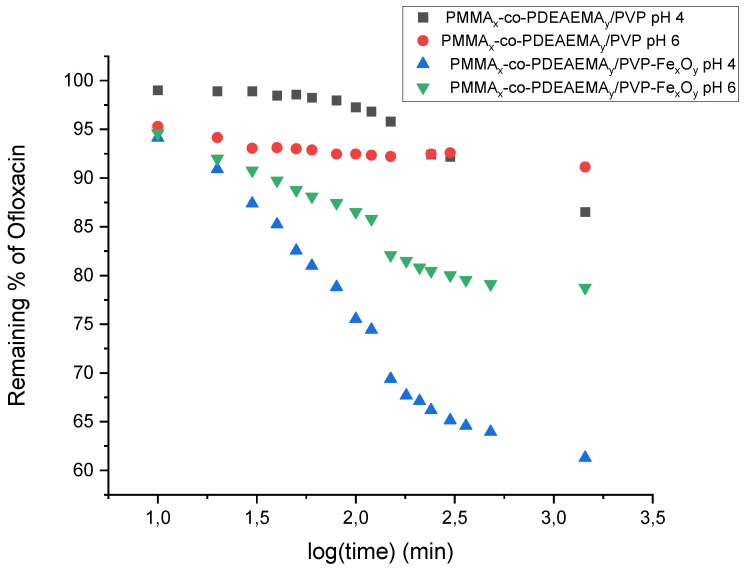
Adsorption kinetic plots for OFL removal recorded in the presence of the pristine (PMMA_x_-co-PDEAEMA_y_/PVP) and magnetoactive (PMMA_x_-co-PDEAEMA_y_/PVP-Fe_x_O_y_) crosslinked electrospun fibrous membranes at pH 4 and 6.

Adsorption kinetic measurements were carried out by immersing the fibrous membranes in OFL-containing aqueous solutions for 24 h and recording the OFL absorbanceat 287 nm at different time intervals after membrane incubation. In order to investigate the adsorption dependency on pH, two different experiments were carried out at pH = 4 and pH = 6. The adsorption kinetic plots corresponding to the two membrane types recorded under the above-mentioned pH conditions are provided in Figure 12.

According to the experimental data provided in Figure 12, the magnetic fibrous adsorbent presented a significantly higher (two- to threefold) adsorption efficiency compared to the non-magnetic analogue at both pH values. More precisely, a 13% and 9% OFL removal was observed in the case of the pristine fibrous membrane at pH 4 and 6, respectively, whereas the percentage removal was increased to 39% and 21% when the magnetically functionalized fibrous adsorbent was used instead. The same positive effect of magnetic nanoparticle functionalization on the adsorption efficacy of previously reported electrospun fibrous membranes employed as substrates for the removal of various contaminants, including antibiotics from aqueous media, was also observed [28,29,60]. Exemplarily, Liu et al. demonstrated the tetracycline removal from aqueous media at a pH range of 4–6, employing a Fe_3_O_4_-functionalized polyacrylonitrile electrospun membrane [60].

The differences observed in the adsorption efficiency of the non-magnetic fibrous membrane at pH 4 and 6 could be attributed to the fact that at pH 4, both the tertiary amino-functionalities in DEAEMA and the OFL amino group were found in the cationic form, which could have resulted in the development of electrostatic repulsive forces that eventually led to lower adsorption efficiency. However, at lower pH, hydrogen bonding interactions existed between the carbonyl group of PVP and the hydrogen of the OFL carboxyl group, which was found in its neutral (non-ionized) form. Consequently, the DEAEMA-OFL repulsive forces could not prevail over the PVP-OFL H-bond interactions, probably due to the smaller percentage of the DEAEMA moieties within the fibrous membrane.

The improved adsorption performance observed in the case of the Fe_x_O_y_ -containing electrospun fibrous membrane, especially at the lowest pH = 4, was attributed to the presence of the Fe_x_O_y_ NPs on the fibers’ surfaces, as revealed by SEM and TEM, that provide additional binding sites for OFL adsorption. Specifically, coordination complexes may form between the carbonyl group of OFL and the hydrous oxide (Fe-OH_2_^+^) that is generated at low pH values [61,62,63].

This result is in line with our group’s previous studies dealing with magnetically functionalized electrospun fibrous membranes that were evaluated as adsorbents for the removal of U(VI) and Eu(III) from aqueous solutions, [28,29] thus highlighting the significance of magnetic functionalization in the development of effective adsorbents destined for use in water-remediation processes.

**Figure 13:**
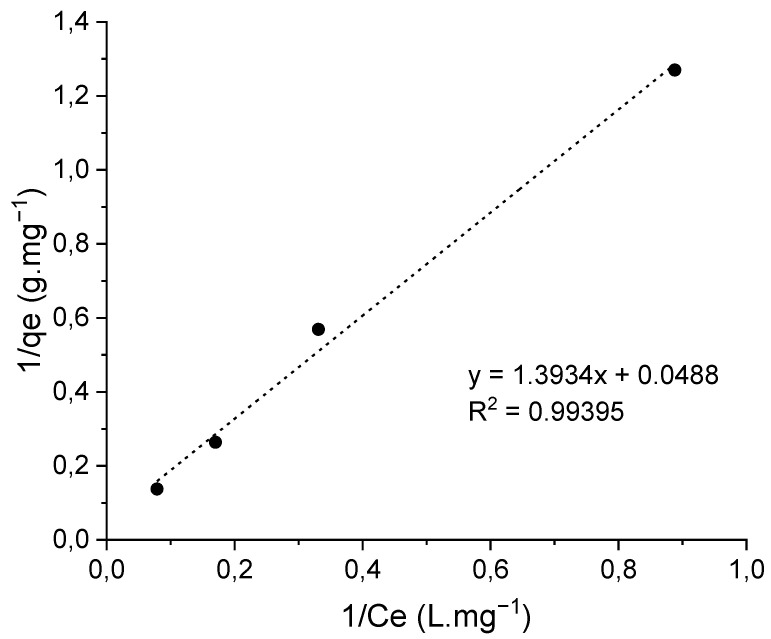
Langmuir isotherm (1/*q_e_* vs. 1/*C_e_*) corresponding to OFL adsorption process, employing the PMMA_x_-co-PDEAEMA_y_/PVP-Fe_x_O_y_ crosslinked fibrous membrane as an adsorbent at pH = 4 (R^2^ = 0.994).

The maximum adsorption capacity (*q_max_*) of the magnetically functionalized fibrous membrane was determined by immersing the adsorbent in aqueous solutions of various OFL concentrations at room temperature and at pH 4 and recording the UV–vis spectrum of the supernatant solution after 24 h. Through use of the absorbance (at 287 nm) vs. OFL concentration calibration curve (provided in Figure 2), the equilibrium concentration of OFL in the solution and the equilibrium adsorbed amount of OFL per unit mass of adsorbent, denoted as *C_e_* (mg·L^−1^) and *q_e_* (mg·g^−1^), respectively, could be determined. By fitting the experimental data to the Langmuir adsorption model expressed mathematically by Equation (4) and plotting 1/*q_e_* versus 1/*C_e_* (Figure 13), the maximum adsorption capacity *q_max_* (mg·g^−1^) and the Langmuir adsorption equilibrium constant *K_d_* (L·mg^−1^) that reflects the adsorption affinity of the binding sites were determined to be 20.5 (mg·g^−1^) and 0.068 (L·mg^−1^), respectively.

(4) $$\frac{1}{q_{e}}=\frac{1}{q_{max}K_{d}C_{e}}+\frac{1}{q_{max}}$$

#### 3.3.2. Removal of OFL from Urban Wastewater

**Figure 14:**
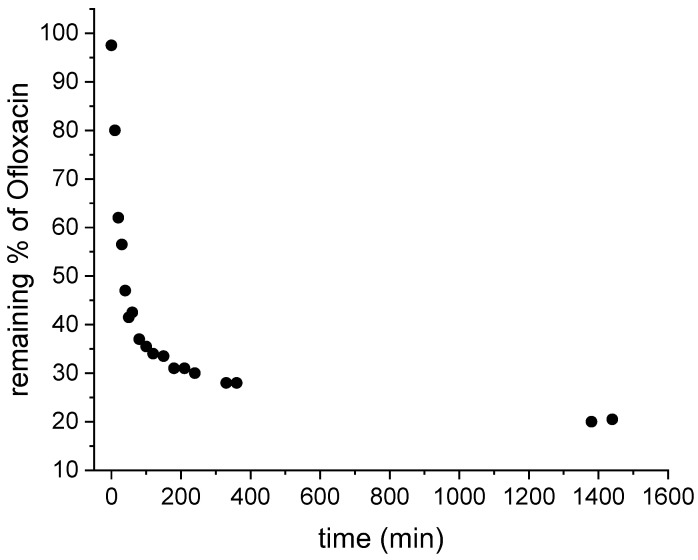
Adsorption kinetic study for OFL-contaminated UWW performed in the presence of the PMMA_x_-*co*-PDEAEMA_y_/PVP/Fe_x_O_y_ crosslinked fibrous membranes at pH 4.

The PMMA_x_-*co*-PDEAEMA_y_/PVP-Fe_x_O_y_ crosslinked fibrous membrane exhibiting the highest adsorption efficiency in the removal of OFL from synthetic aqueous solutions at pH = 4 was selected to be further evaluated as a substrate for the removal of OFL from secondary urban wastewater (UWW). The latter was spiked with OFL at a concentration of 2.34 mg·L^−1^. By recording the characteristic absorbancesignal of free (unbound) OFL appearing at 287 nm at different time intervals after membrane incubation, the % remaining of OFL vs. time plot could be constructed (Figure 14), demonstrating a high removal efficiency by reaching 80% after 24 h incubation time.

### 3.4. Desorption Studies

**Figure 15:**
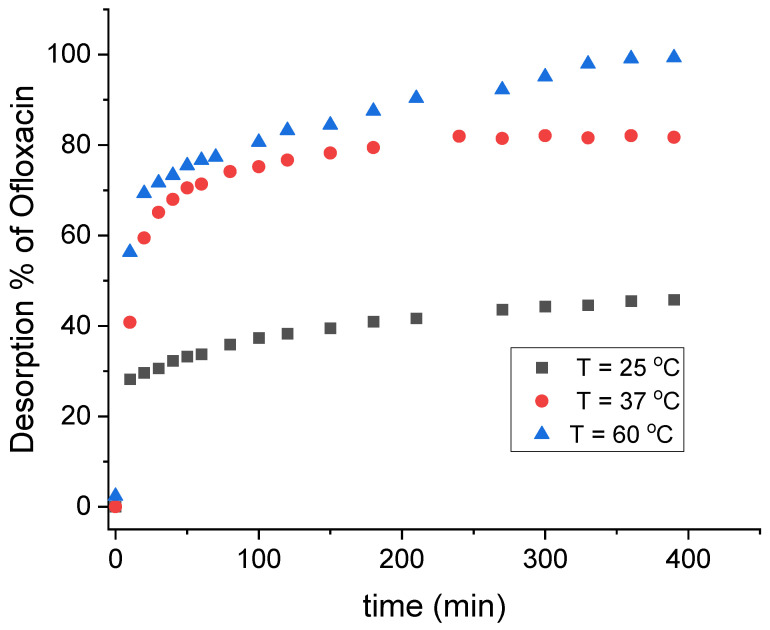
OFL % desorption versus time plot recorded upon immersing the OFL-containing PMMA_x_-*co*-PDEAEMA_y_/PVP/Fe_x_O_y_ crosslinked fibrous membrane in an alkaline aqueous solution at various temperatures.

Desorption of the adsorbed OFL was realized by immersing the OFL-containing magnetoactive fibrous membrane in alkali solutions at various temperatures. The desorption profile of OFL recorded at 25 °C, 37 °C and 60 °C is provided in Figure 15. As seen, a rapid OFL release was observed within the first 10 min in all cases while temperature was found to play a significant role in the desorption process, since desorption % of 46, 82 and 100 were recorded at 25 °C, 37 °C and 60 °C, respectively. This result agrees with a previous study by Mohhamad et al. in which the authors stated that an increase in temperature may raise the kinetic energy of the molecules, resulting in the gaining of higher energy than that required for the adsorption process to occur [64].

## 4. Conclusions

Magnetically functionalized electrospun microfibrous membranes containing tertiary amino functionalities were fabricated and evaluated as adsorbents for the removal of the antibiotic Ofloxacin from synthetic aqueous media and urban wastewater. To further increase the adsorption efficiency and at the same time impart magnetic properties to the produced fibrous membranes, the latter underwent a post-magnetization step resulting in the anchoring of magnetic iron oxide NPs onto the fibers’ surfaces. SEM and TEM verified the anchoring of Fe_x_O_y_ NPs onto the fibers’ surfaces while VSM was used to study the magnetic properties of the produced fibrous nanocomposites. The latter were found to be superparamagnetic, exhibiting a high Ms value (12.3 emu/g) that corresponded to ~13% wt. magnetic loading. Moreover, through employment of a magnetization theoretical model, a diameter of 5.4 ± 2.3 nm was calculated for the produced magnetic NPs that were deposited onto the fibrous membranes during the post-magnetization step. Furthermore, magnetic functionalization resulted in the mechanical enhancement under tensile loading conditions, since the magnetically functionalized adsorbents exhibited higher Young’s modulus and higher yield stress in comparison to the pristine polymer analogues.

The adsorption capacity of the magnetically functionalized nanocomposite fibrous adsorbents was evaluated as a function of various physicochemical parameters, including the initial OFL solution concentration, the solution pH and the magnetic loading. More precisely, adsorption kinetic measurements were carried out at pH = 4 and pH = 6. In both cases, the magnetic fibrous adsorbent presented a significantly higher (two- to threefold) adsorption efficiency compared to the non-magnetic analogue while the adsorption was more effective at pH 4. Hence, to determine the maximum adsorption capacity (*q_max_*) of the magnetically functionalized fibrous membrane, the latter was immersed in aqueous solutions of various OFL concentrations at pH 4. By fitting of the experimental data to the Langmuir adsorption model, the maximum adsorption capacity *q_max_* (mg·g^−1^) and the Langmuir adsorption equilibrium constant *K_d_* (L·mg^−1^) were determined to be 20.5 (mg·g^−1^) and 0.068 (L·mg^−1^), respectively.

The magnetically functionalized nanocomposite fibrous membrane exhibiting the highest adsorption efficiency in the removal of OFL from synthetic aqueous solutions at pH = 4 was successfully evaluated as a substrate for the removal of OFL from secondary urban wastewater, reaching 80% removal efficiency after 24 h. Finally, it was demonstrated that OFL desorption could be realized upon exposing the OFL-loaded at elevated temperatures.

## Data Availability

Not applicable.

## References

[1] Chinnaiyan P., Thampi S.G., Kumar M., Mini K.M. (2018). Pharmaceutical products as emerging contaminant in water: Relevance for developing nations and identification of critical compounds for Indian environment. Environ. Monit. Assess..

[2] Gonzalez Pena O.I., Lopez Zavala M.A., Ruelas H.C. (2021). Pharmaceuticals Market, Consumption Trends and Disease Incidence Are Not Driving the Pharmaceutical Research on Water and Wastewater. Int. J. Environ. Res. Public Health.

[3] Abd El-Monaem E.M., Eltaweil A.S., Elshishini H.M., Hosny M., Abou Alsoaud M.M., Attia N.F., El-Subruiti G.M., Omer A.M. (2022). Sustainable adsorptive removal of antibiotic residues by chitosan composites: An insight into current developments and future recommendations. Arab. J. Chem..

[4] Gonsioroski A., Mourikes V.E., Flaws J.A. (2020). Endocrine Disruptors in Water and Their Effects on the Reproductive System. Int. J. Mol. Sci..

[5] Monarca S., Feretti D., Collivignarelli C., Guzzella L., Zerbini I., Bertanza G., Pedrazzani R. (2000). The influence of different disinfectants on mutagenicity and toxicity of urban wastewater. Water Res..

[6] Rosal R., Rodríguez A., Perdigón-Melón J.A., Petre A., García-Calvo E., Gómez M.J., Agüera A., Fernández-Alba A.R. (2010). Occurrence of emerging pollutants in urban wastewater and their removal through biological treatment followed by ozonation. Water Res..

[7] Priya A.K., Gnanasekaran L., Rajendran S., Qin J., Vasseghian Y. (2022). Occurrences and removal of pharmaceutical and personal care products from aquatic systems using advanced treatment-A review. Environ. Res..

[8] Eniola J.O., Kumar R., Barakat M.A., Rashid J. (2022). A review on conventional and advanced hybrid technologies for pharmaceutical wastewater treatment. J. Clean. Prod..

[9] Ullaha A., Shahzadab K., Wali Khanc S., Starov V. (2020). Purification of produced water using oscillatory membrane filtration. Desalination.

[10] Kosutic K., Dolar D., Asperger D., Kunst B. (2007). Removal of antibiotics from a model wastewater by RO/NF membranes. Sep. Purif. Technol..

[11] Michael I., Rizzo L., McArdell C.S., Manaia C.M., Merlin C., Schwartz T., Dagot C., Fatta-Kassinos D. (2013). Urban wastewater treatment plants as hotspots for the release of antibiotics in the environment: A review. Water Res..

[12] Chakraborty R., Asthanaa A., Kumar Singh A., Jaina B., Hasan Susan A.B. (2022). Adsorption of heavy metal ions by various low-cost adsorbents: A review. J. Environ. Anal. Chem..

[13] Zhu F., Zheng Y.M., Zhang B.G., Dai Y.R. (2021). A critical review on the electrospun nanofibrous membranes for the adsorption of heavy metals in water treatment. J. Hazard. Mater..

[14] Nayl A.A., Abd-Elhamid A.I., Awwad N.S., Abdelgawad M.A., Wu J., Mo X., Gomha S.M., Aly A.A., Bräse S. (2022). Review of the Recent Advances in Electrospun Nanofibers Applications in Water Purification. Polymers.

[15] Uddin Z., Ahmad F., Ullan T., Nawab Y., Ahmad S., Azam F., Rasheed A., Zafar M.S. (2022). Recent trends in water purifcation using electrospun nanofbrous membranes. Int. J. Environ. Sci. Technol..

[16] Almasian A., Olya M.E., Mahmoodi N.M. (2015). Synthesis of polyacrylonitrile/polyamidoamine composite nanofibers using electrospinning technique and their dye removal capacity. J. Taiwan Inst. Chem. Eng..

[17] Xu Y., Bao J., Zhang X., Li W., Xie Y., Sun S., Zhao W., Zhao C. (2019). Functionalized polyethersulfone nanofibrous membranes with ultra-high adsorption capacity for organic dyes by one-step electrospinning. J. Colloid Interface Sci..

[18] Zhao K., Kang S.X., Yang Y.Y., Yu D.G. (2021). Electrospun Functional Nanofiber Membrane for Antibiotic Removal in Water: Review. Polymers.

[19] Papaphilippou P.C., Vyrides I., Mpekris F., Stylianopoulos T., Papatryfonos C.A., Theocharis C.R., Krasia-Christoforou T. (2015). Evaluation of novel, cationic electrospun microfibrous membranes as adsorbents in bacteria removal. RSC Adv..

[20] Fahimirad S., Fahimirad Z., Sillanpää M. (2021). Efficient removal of water bacteria and viruses using electrospun nanofibers. Sci. Total. Environ..

[21] Vass P., Szabó E., Domokos A., Hirsch E., Galata D., Farkas B., Démuth B., Andersen S.K., Vigh T., Verreck G. (2020). Scale-up of electrospinning technology: Applications in the pharmaceutical industry. WIREs Nanomed. Nanobiotechnol..

[22] Omer S., Forgách L., Zelkó R., Sebe I. (2021). Scale-up of Electrospinning: Market Overview of Products and Devices for Pharmaceutical and Biomedical Purposes. Pharmaceutics.

[23] Zhang C.L., Yu S.H. (2014). Nanoparticles meet electrospinning: Recent advances and future prospects. Chem. Soc. Rev..

[24] Arrieta M.P., Gil A.L., Yusef M., Kenny J.M., Peponi L.P. (2020). Electrospinning of PCL-Based Blends: Processing Optimization for Their Scalable Production. Materials.

[25] Homocianu M., Pascariu P. (2020). Electrospun Polymer-Inorganic Nanostructured Materials and Their Applications. Poly. Rev..

[26] Patel S., Hota G. (2016). Iron oxide nanoparticles immobilized PAN nanofibers: Synthesis and adsorption studies. RSC Adv..

[27] Alharbi H.F., Haddad M.Y., Aijaz M.O., Assaifan A.K., Kari M.R. (2020). Electrospun Bilayer PAN/Chitosan Nanofiber Membranes Incorporated with Metal Oxide Nanoparticles for Heavy Metal Ion Adsorption. Coatings.

[28] Philippou K., Christou C.N., Socoliuc V., Vekas L., Tanasă E., Miclau M., Pashalidis I., Krasia-Christoforou T. (2021). Superparamagnetic polyvinylpyrrolidone/chitosan/Fe_3_O_4_ electrospun nanofibers as effective U(VI) adsorbents. J. Appl. Polym. Sci..

[29] Panteli S., Savva I., Efstathiou M., Vekas L., Marinica O.M., Krasia-Christoforou T., Pashalidis I. (2019). β-Ketoester-Functionalized Magnetoactive Electrospun Polymer Fibers as Eu (III) Adsorbents. SN Appl. Sci..

[30] Zhao R., Li X., Li Y., Li Y., Sun B., Zhang N., Chao S., Wang C. (2017). Functionalized magnetic iron oxide/polyacrylonitrile composite electrospun fibers as effective chromium (VI) adsorbents for water purification. J. Colloid Interface Sci..

[31] Torasso N., Vergara-Rubio A., Rivas-Rojas P., Huck-Iriart C., Larrañaga A., Fernández-Cirelli A., Cerveny S., Goyanes S. (2021). Enhancing arsenic adsorption via excellent dispersion of iron oxide nanoparticles inside poly(vinyl alcohol) nanofibers. J. Environ. Chem. Eng..

[32] Savva I., Marinica O., Papatryfonos C.A., Vekas L., Krasia-Christoforou T. (2015). Evaluation of electrospun polymer–Fe_3_O_4_ nanocomposite mats in malachite green adsorption. RSC Adv..

[33] Liu Q., Zheng Y., Zhong L., Cheng X. (2015). Removal of tetracycline from aqueous solution by a Fe_3_O_4_ incorporated PAN electrospun nanofiber mat. J. Environ. Sci..

[34] Mukhortova Y.R., Pryadko A.S., Chernozem R.V., Pariy I.O., Akoulina E.A., Demianova I.V., Zharkova I.I., Ivanov Y.F., Wagner D.V., Bonartsev A.P. (2022). Fabrication and characterization of a magnetic biocomposite of magnetite nanoparticles and reduced graphene oxide for biomedical applications. Nano-Struct. Nano-Objects.

[35] Joshi N.C., Gururani P. (2022). Advances of graphene oxide based nanocomposite materials in the treatment of wastewater containing heavy metal ions and dyes. Curr. Opin. Green Sustain. Chem..

[36] Mura S., Jiang Y., Vassalini I., Gianoncelli A., Alessandri I., Granozzi G., Calvillo L., Senes N., Enzo S., Innocenzi P. (2018). Graphene Oxide/Iron Oxide Nanocomposites for Water Remediation. ACS Appl. Nano Mater..

[37] Mong Thy L.T., Hoai Thuong N., Hoang Tu T., Minh Nam H., Huu Hieu N., Thanh Phong M. (2019). Synthesis of magnetic iron oxide/graphene oxide nanocomposites for removal of cadmium ions from water. Adv. Nat. Sci. Nanosci. Nanotechnol..

[38] Su H., Ye Z., Hmid N. (2017). High-performance iron oxide-graphene oxide nanocomposite adsorbents for arsenic removal. Colloids Surf. A Physicochem. Eng. Asp..

[39] Mong Thya L.T., Mai Cuong P., Hoang Tua T., Minh Nam H., Huu Hieua N., Thanh Phong M. (2020). Fabrication of Magnetic Iron Oxide/Graphene Oxide Nanocomposites for Removal of Lead Ions from water. Chem. Eng. Trans..

[40] Samie B., Toosi A. (2010). Adsorption of malachite green on silica gel: Effects of NaCl, pH and 2-propanol. J. Hazard. Mater..

[41] Liu X., Xu Y., Yu J., Li S., Wang J., Wang C., Chu F. (2014). Integration of lignin and acrylic monomers towards grafted copolymers by free radical polymerization. Int. J. Biol. Macromol..

[42] Christou C., Philippou K., Krasia-Christoforou T., Pashalidis I. (2019). Uranium adsorption by polyvinylpyrrolidone/chitosan blended nanofibers. Carbohydr. Polym..

[43] Safarik I., Pospiskova K., Baldikova E., Savva I., Vekas L., Marinica O., Tanasa E., Krasia-Christoforou T. (2018). Fabrication and Bioapplications of Magnetically Modified Chitosan-based Electrospun Nanofibers. Electrospinning.

[44] Huang S.J., Ke J.H., Chen G.J., Wang L.F. (2013). One-pot synthesis of PDMAEMA-bound iron oxide nanoparticles for magnetofection. J. Mater. Chem. B..

[45] Bhattarai N., Edmondson D., Veiseh O., Matsen F.A., Zhang M. (2005). Electrospun chitosan-based nanofibers and their cellular compatibility. Biomaterials.

[46] Savva I., Efstathiou M., Krasia-Christoforou T., Pashalidis I. (2013). Adsorptive removal of U(VI) and Th(IV) from aqueous solutions using polymer-based electrospun PEO/PLLA fibrous membranes. J. Radioanal. Nucl. Chem..

[47] Starodubtsev S.G., Saenko E.V., Dokukin M.E., Aksenov V.L., Klechkovskaya V.V., Zanaveskina I.S., Khokholov A.R. (2005). Formation of magnetite nanoparticles in poly(acrylamide) gels. J. Phys. Cond. Matter.

[48] Gong T., Yang D., Hu J., Yang W., Wang C., Lu J.Q. (2009). Preparation of monodispersed hybrid nanospheres with high magnetite content from uniform Fe_3_O_4_ clusters. Colloids Surf. A Physicochem. Eng. Aspects.

[49] Wan S., Zheng Y., Liu Y., Yan H., Liu K. (2005). Fe_3_O_4_ nanoparticles coated with homopolymers of glycerol mono(meth)acrylate and their bloc copolymers. J. Mater. Chem..

[50] Papaphilippou P.C., Pourgouris A., Marinica O., Taculescu A., Athanasopoulos G.I., Vekas L., Krasia-Christoforou T. (2011). Fabrication and characterization of superparamagnetic and thermoresponsive hydrogels based on oleic-acid-coated Fe_3_O_4_ nanoparticles, hexa(ethylene glycol) methyl ether methacrylate and 2-(acetoacetoxy)ethyl methacrylate. J. Magn. Magn. Mater..

[51] Ivanov A.O., Kantorovich S.S., Reznikov E.N., Holm C., Pshenichnikov A.F., Lebedev A.V., Chremos A., Camp P.J. (2007). Magnetic properties of polydisperse ferrofluids: A critical comparison between experiment, theory, and computer simulation. Phys. Rev. E Stat. Nonlin. Soft Matter Phys..

[52] Tolmacheva V.V., Apyari V.V., Ibragimova B.N., Kochuk E.V., Dmitrienko S.G., Zolotov Y.A. (2015). A Polymeric Magnetic Adsorbent Based on Fe_3_O_4_ Nanoparticles and Hypercrosslinked Polystyrene for the Preconcentration of Tetracycline Antibiotics. J. Anal. Chem..

[53] Teng Y., Li Y., Li Y., Song Q. (2020). Preparation of Fe3O4/PVP magnetic nanofibers via in situ method with electrospinning. J. Phys. Conf. Ser..

[54] Sepulveda-Guzman S., Lara L., Perez-Camacho O., Rodrıguez-Fernandez O., Olivas A., Escudero R. (2007). Synthesis and characterization of an iron oxide poly(styreneco-carboxybutylmaleimide) ferrimagnetic composite. Polymer.

[55] Zhu C., Lang Y., Liu B., Zhao H. (2019). Ofloxacin Adsorption on Chitosan/Biochar Composite: Kinetics, Isotherms, and Effects of Solution Chemistry. Polycycl. Aromat. Compd..

[56] Vojnovic B., Cetina M., Franjkovic P., Sutlovic A. (2022). Influence of Initial pH Value on the Adsorption of Reactive Black 5 Dye on Powdered Activated Carbon: Kinetics, Mechanisms, and Thermodynamics. Molecules.

[57] Li H., Zhang D., Han X., Xing B. (2014). Adsorption of antibiotic ciprofloxacin on carbon nanotubes: pH dependence and thermodynamics. Chemosphere.

[58] Ji L., Chen W., Zheng S., Xu Z., Zhu D. (2009). Adsorption of Sulfonamide Antibiotics to Multiwalled Carbon Nanotubes. Langmuir.

[59] Van Wieren E.M., Seymour M.D., Peterson J.W. (2012). Interaction of the fluoroquinolone antibiotic, ofloxacin, with titanium oxide nanoparticles in water: Adsorption and breakdown. Sci. Total Environ..

[60] Liu Q., Zhong L.B., Zhao Q.B., Frear C., Zheng Y.M. (2015). Synthesis of Fe_3_O_4_/Polyacrylonitrile Composite Electrospun Nanofiber Mat for Effective Adsorption of Tetracycline. ACS Appl. Mater. Interfaces.

[61] Gu C., Karthikeyan K.G. (2005). Interaction of Tetracycline with Aluminum and Iron Hydrous Oxides. Environ. Sci. Technol..

[62] Streat M., Newton N.L.R. (2008). Hydrous ferric oxide as an adsorbent in water treatment: Part 1. Preparation and physical characterization. Process Saf. Environ. Prot..

[63] Li Y., Gao L., Lu Z., Wang Y., Wang Y., Wan S. (2020). Enhanced Removal of Heavy Metals from Water by Hydrous Ferric Oxide-Modified Biochar. ACS Omega.

[64] Mohammad N., Atassi Y. (2020). Adsorption of methylene blue onto electrospun nanofbrous membranes of polylactic acid and polyacrylonitrile coated with chloride doped polyaniline. Sci. Rep..

